# A multimode photodetector with polarization-dependent near-infrared responsivity using the tunable split-dual gates control

**DOI:** 10.1016/j.isci.2022.105164

**Published:** 2022-09-19

**Authors:** Zhou Zhang, Junxin Chen, Hao Jia, Jianfa Chen, Feng Li, Ximiao Wang, Shaojing Liu, Hai Ou, Song Liu, Huanjun Chen, Ya-Qing Bie, Shaozhi Deng

**Affiliations:** 1 School of Electronics and Information Technology, Sun Yat-sen University, Guangzhou 510006, China; 2 State Key Lab of Optoelectronic Materials and Technologies, Guangdong Province Key Laboratory of Display Material and Technology, Sun Yat-sen University, Guangzhou 510006, China; 3 Shenzhen Institute for Quantum Science and Engineering, Southern University of Science and Technology, Shenzhen 518055, China

**Keywords:** Optical materials, Nanomaterials, Electronic materials

## Abstract

As the limited carrier densities in atomic thin materials can be well controlled by electrostatic gates, p-n junctions based on two-dimensional materials in the coplanar split-gate configuration can work as photodetectors or light-emitting diodes. These coplanar gates can be fabricated in a simple one-step lithography process and are frequently used in hybrid integration with on-chip optical structures. However, the polarization-dependent responsivity of such a configuration is less explored in the near-infrared band, and a clear understanding is still missing. Here we fabricate near-infrared tunable multiple modes twisted bilayer graphene photodetector enabled by the coplanar split-gate control and confirm that the photothermoelectric effect governs the photovoltage mechanism of the p-n junction mode. Our study also elucidates that the discrepancy of the responsivities under different linear polarizations is owing to the different cavity modes and provides a valuable example for designing chip-integrated optoelectronic devices.

## Highlights

• Design of coplanar split-gated controlled multimode near-infrared photodetector
• Verification of the photothermoelectric mechanism of the p-n junction
• Understanding the reason for the polarization-dependent responsivity

## Graphical abstract

**Figure:**
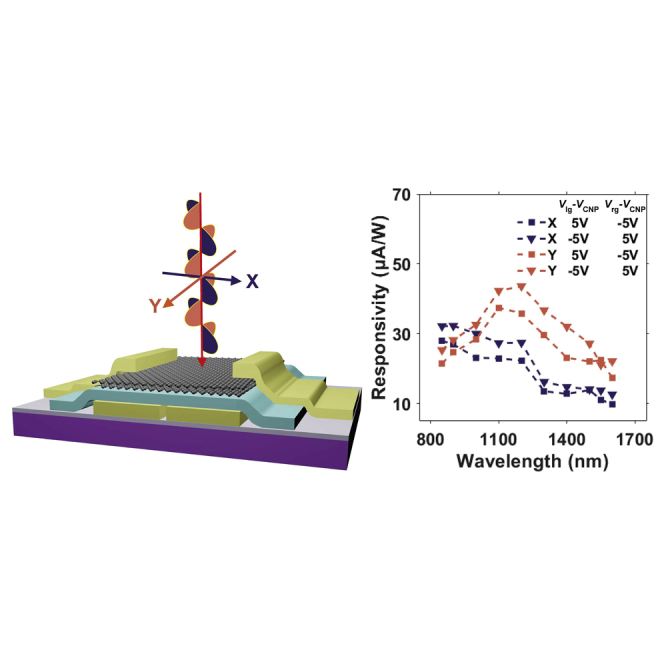

## Introduction

Carrier density in semiconductors is one of the most crucial parameters for functional devices such as various field-effect transistors, p-n junctions, and so forth. In bulk materials, only a small proportion of carriers, i.e., the surface charge densities can be tuned by an electrostatic gate. But for two-dimensional materials, carriers in the atomic thin layers can be fully controlled via gating, and many functional devices have been developed with different gate controls (Mak et al., 2013; Saito et al., 2016; Schwierz, 2010). For example, the top/bottom dual gates (Ju et al., 2017; Ma et al., 2014) are often used because they can independently tune the charge density as well as the electric field strength within the active material. While the fabrication of both top and bottom gates requires multiple lithography processes at different layers, coplanar gates can be fabricated in the same lithography process. Coplanar gates have been used to define active areas in devices, such as arc-shaped gates (Petta et al., 2005) for creating quantum dots in semiconductors, ring-shaped electrodes for controlling field emitters (Zhao et al., 2018), and split-gates for making narrow p-n junctions (de Vries et al., 2021). Especially, for narrow line-shape p-n junctions, which are often used as light emitters (Bie et al., 2017; Ross et al., 2014) and high-speed photodetectors (Muench et al., 2019; Schuler et al., 2018), the coplanar split-gate design is convenient for the hybrid integration with on-chip optical structures such as slot (Schuler et al., 2016) or photonic waveguides (Bie et al., 2017; Schuler et al., 2018). However, for p-n junction photodetectors in the split-gate configuration, less attention has been paid to the polarization-dependent responsivity which can be modified by the field enhancement effect owing to the cavity mode in the near-infrared range. Such effectsthe one side of the can be neglected for photodetectors in the far-infrared region (Herring et al., 2014) as the gap size of the gold split-gate is usually only hundreds of nanometers. However, it is substantial in the visible-near IR range.

Here we fabricate the twisted bilayer graphene (tBLG) photodetector in the coplanar split-gate configuration. The device can perform in multiple modes as field-effect transistor, Schottky junction, and p-n junction photodetector under different electrostatic gate voltages. We confirmed that the photothermoelectric effect governs the photovoltage mechanism of the p-n junction. This study also elucidates the discrepancy of the near-infrared photodetector responsivity under different linear polarizations in the split-gate p-n junction configuration and helps the design of active two-dimensional optoelectronic devices.

## Results and discussion

**Figure 1:**
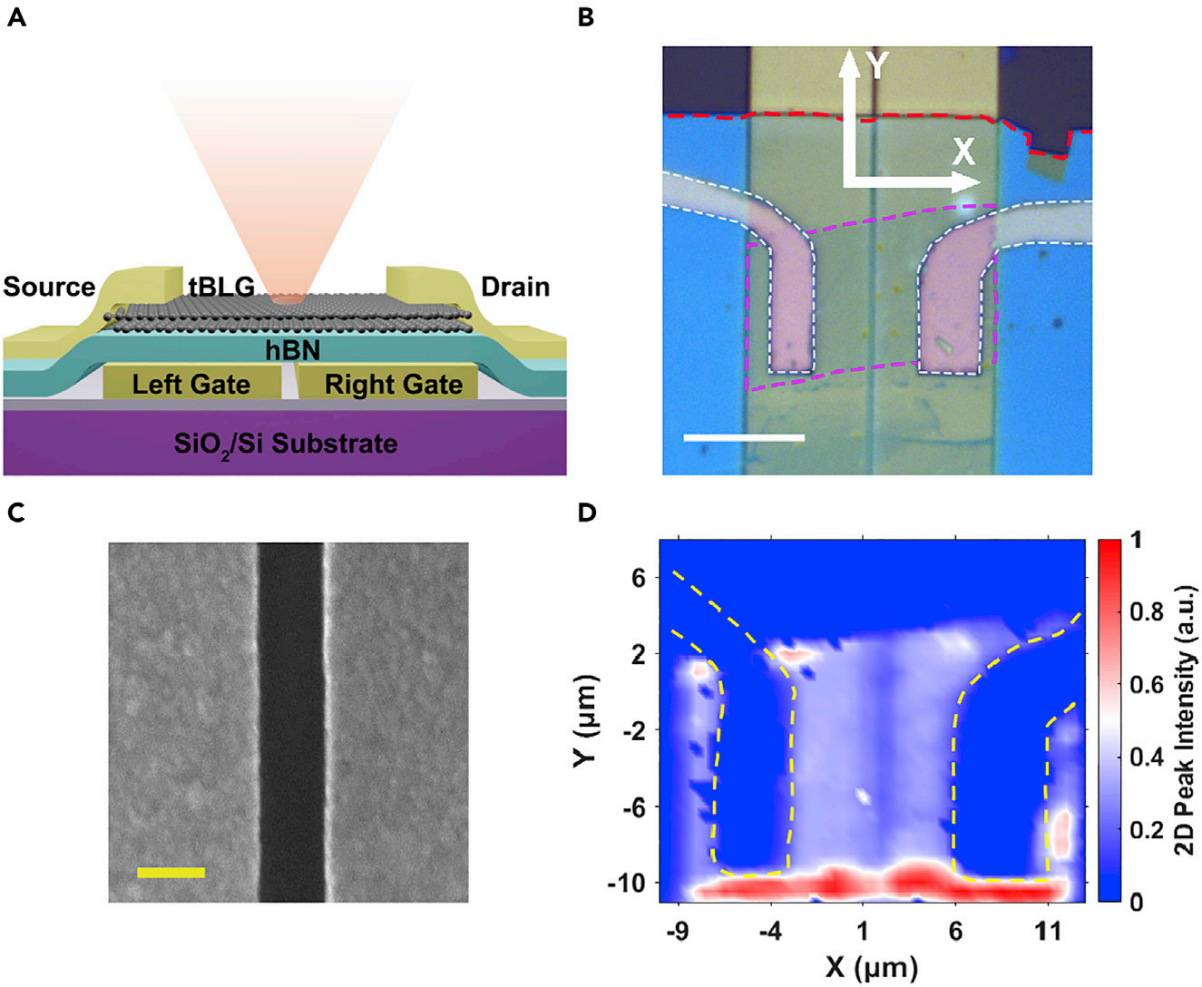
The design of a multimode split-gate device (A) The schematic structure of the device with tunable dual gates; the gap size between the left and the right gate is 200 nm; an h-BN flake is used as the dielectric layer. (B) Optical image of a real device. The scale bar is 10 μm and the pink dashed line surrounds the tBLG area and the red dashed line indicates the edge of a 30 nm thick h-BN flake. (C) The SEM image of the Au film gap and the scale bar is 200 nm. (D) Raman mapping of the device using the 2,693 cm^−1^ peak of the graphene excited by a 532 nm laser. The bright region is mainly the twisted graphene region while the intense red color indicates the curled graphene edges.

As a broadband absorber, graphene has been investigated as an active material for photodetectors in terahertz (Bandurin et al., 2018), infrared (Guo et al., 2018), and visible range (Tang et al., 2020). In principle, tBLG with a twist angle around 4.5° also has a flat broadband response but a weak enhancement of absorption (Yu et al., 2019)near 1,400 nm owing to the van Hove singularity (Brihuega et al., 2012). Figure 1A depicts the design of the device. The tBLG is placed on a hexagonal-Boron nitride (h-BN) dielectric layer using the transfer method (Zomer et al., 2014). The multilayer stack is placed on the split gold film gates which can independently induce the electron or hole doping. The source and drain electrodes were made of 1 nm/35 nm Cr/Au film by electron beam lithography and thermal evaporation. According to previous studies (Giubileo and Di Bartolomeo, 2017), the contact resistance will be much larger if the thickness of the sticky layer chromium is larger than 1 nm. The split-gates are prepatterned on SiO_2_/Si using the lithography method. The optical image of the real device is shown in Figure 1B. The air gap between the gold gates is visible and the gap size is confirmed to be 200 nm using an SEM image as shown in Figure 1C. If a larger gap such as 400 nm is used, the device is more like a p-i-n junction instead of a p-n junction and if the gap is reduced to less than 100 nm, the possibility of leak current between the gate electrodes will increase. To better identify the tBLG region, we took a Raman mapping measurement using the graphene 2D peak (2,693 cm^−1^) and the result is shown in Figure 1D. The Raman spectrum of the tBLG region is shown in Figure S1.

**Figure 2:**
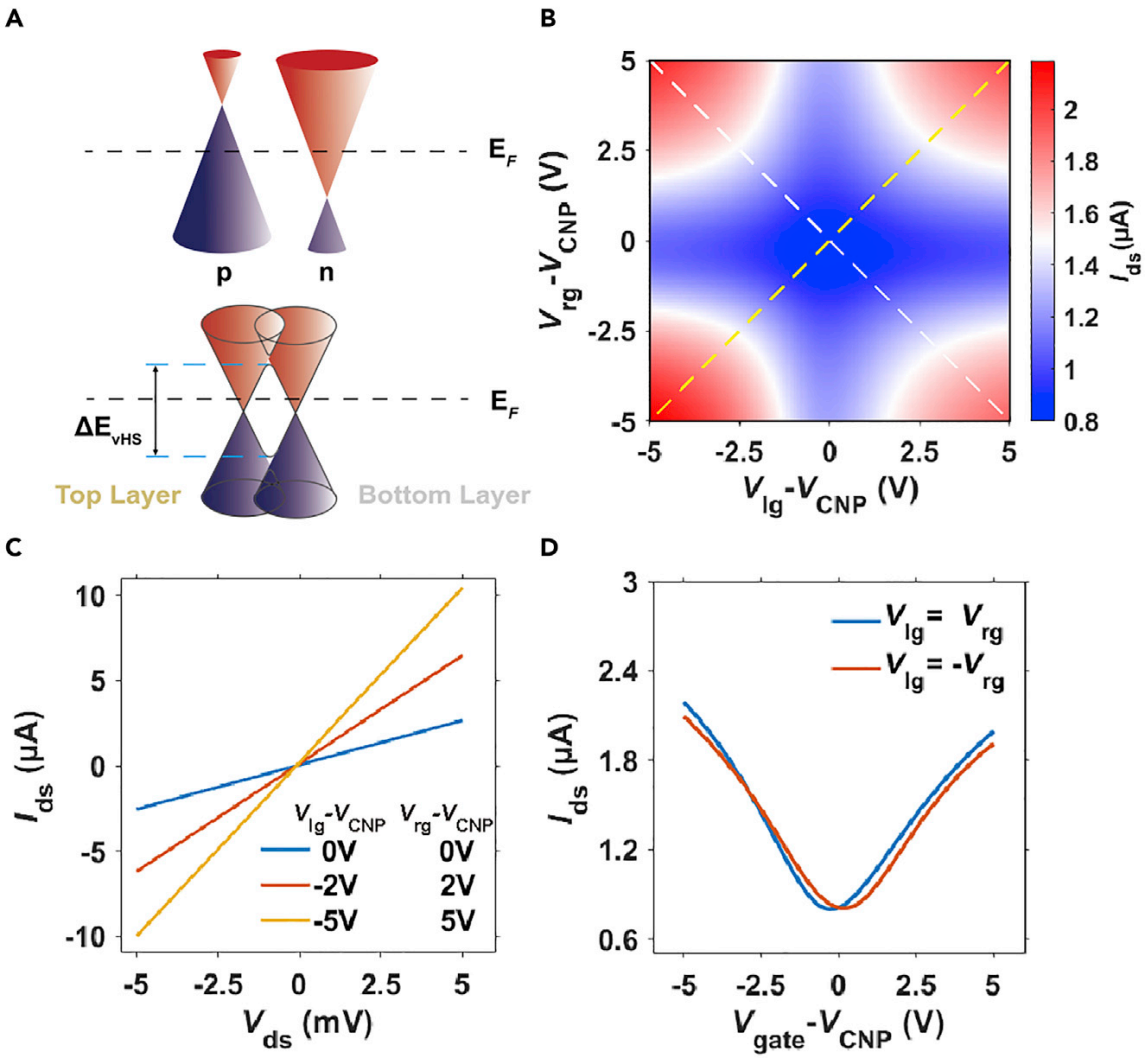
Electrical properties of the device (A) band alignment with different gate controls; top panel: p-n junction; bottom panel: transistor mode. (B) The current-gate voltages relation at dark with *V*_ds_ = 1 mV; *V*_lg_ = *V*_rg_ (the yellow dashed line) and *V*_lg_ = – *V*_rg_ (the white dashed line). (C) The current-voltage (*I*_ds_-*V*_ds_) curves at different gate voltages; p-n junction: *V*_lg_–*V*_cnp_ = *V*_cnp_ –*V*_rg_ = −5 V or −2 V; (D) the dashed line cuts in (B) (*I*_ds_-*V*_g_ relation) at *V*_lg_ = *V*_rg_ and *V*_lg_ = – *V*_rg_.

After the fabrication, the electrical properties of the device were measured in a vacuum (5 × 10^−5^ Pa) cryostat at 77 K because the device stability will be improved at low temperatures and we also provide some results measured at room temperature in Figure S5. Figure 2A shows the band alignment of the p-n junction in the upper panel and the band alignment of the field-effect transistor mode in the bottom panel. Figure 2B is the current –gate voltages (*I*_ds_-*V*_lg_, *V*_rg_) relation at dark with *V*_ds_ = 1 mV. Here we use the subscript “lg” and “rg” to represent the left gate and the right gate. Along the diagonal direction marked by the yellow dashed line, the device is in the bipolar field-effect transistor mode and both sides are doped with the same type of carriers. When the *V*_ds_ is fixed at 1 mV and the gate voltages (*V*_lg_–*V*_cnp_ = *V*_rg_– *V*_cnp_) change from negative to positive, the transfer curve is shown in Figure 2D. The *V*_cnp_ represents the gate voltage that set the Fermi level of graphene near the Dirac point which is also called the charge neutrality point. By using the direct transconductance method (Zhong et al., 2015), the field-effect mobility is estimated to be 3,917 cm^2^/V·s and 2,869 cm^2^/V·s for hole doping and electron doping side. Along the off-diagonal direction which is marked by the white dashed line in Figure 2B, the device is in either a p-n junction or n-p junction mode and the two sides are doped with the opposite type of carriers. Unlike the traditional semiconductor p-n junction, the graphene p-n junction conducts well with both positive and negative bias owing to the Klein tunneling (Katsnelson et al., 2006). Because of the tunneling behavior, the drain-source current-voltage (*I*_ds_*-V*_ds_) relations are linear for the p-n or n-p junction as shown in Figure 2C, and the *I*_ds_*-V*_lg_, *V*_rg_ relation is plotted as the red curve in Figure 2D. All these electrical measurements confirm the p-n junction and the transistor modes controlled by the coplanar split-gates.

**Figure 3:**
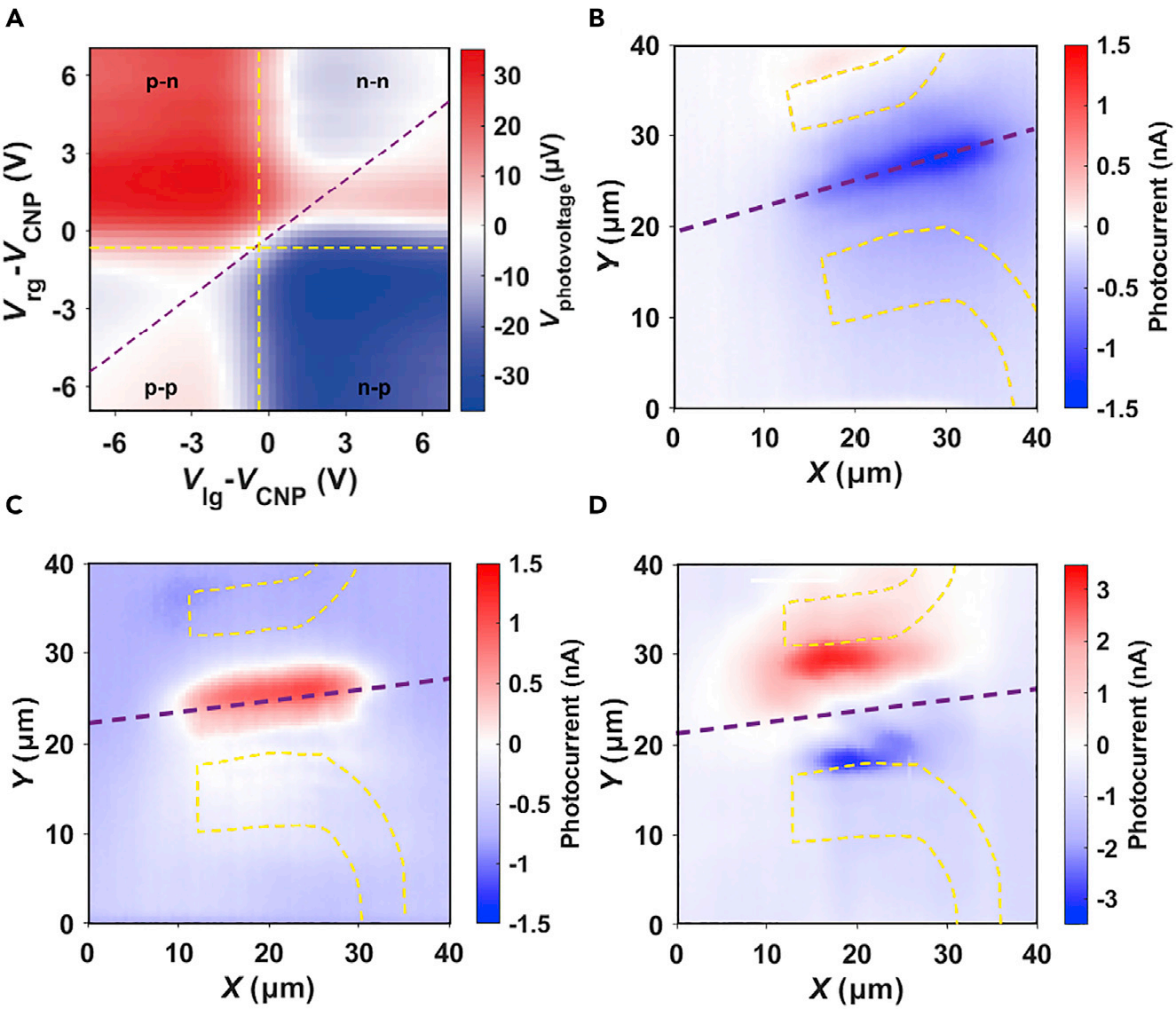
Optical response of the device (A) Photovoltage (*V*_ph_) versus *V*_lg_ and *V*_rg_ at T = 77 K, measured at the junction area. The purple dashed line and the yellow dashed lines mark the region where the sign of the photovoltage switches. (B–D) Photocurrent mappings for the p-n, n-p junction, and charge neutrality condition; the junction position is marked by the purple dashed lines and the position is calibrated with the reflection image collected simultaneously. (B) *V*_lg_ −*V*_cnp_ = − (*V*_rg_−*V*_cnp_) = − 5 V; (C) *V*_lg_ −*V*_cnp_ = − (*V*_rg_−*V*_cnp_) = 5 V; (D) *V*_lg_ −*V*_cnp_ = − (*V*_rg_−*V*_cnp_) = 0 V. The incident laser wavelength is l = 850 nm and the optical power is 1 μW in (a) and 30 μW in (B-D).

To characterize the generated photovoltage of the photodetector, the device is biased at zero voltage and the incident light is focused on the p-n junction via a microscopic confocal system as shown in Figure S3A. The photovoltage between the source-drain leads can be measured using the lock-in technique. Figure 3A shows the generated photovoltage when the 850 nm laser is focused at the gap area and the split-gate voltages are tuned between −7 and 7 V. The change in the photovoltage sign marked by the dashed lines makes the colormap into a 6-fold pattern. As demonstrated in monolayer graphene, the photovoltage is determined by the photothermoelectric effect (Gabor et al., 2011; Herring et al., 2014; Ma et al., 2014). The sign and magnitude of the photovoltage depend on the Seebeck coefficient on each side of the junction (Gabor et al., 2011; Herring et al., 2014; Ma et al., 2014) and can be written as

(Equation 1) $$V_{\text{ph}}=\left(S_{\text{l}}-S_{\text{r}}\right)\nabla T$$

where *S*_l_ (*S*_r_) is the Seebeck coefficient at the left (right) side and the ∇ *T* is the temperature gradient between the laser excitation position and the surrounding area. According to the Mott formula, the Seebeck coefficient *S* is written as

(Equation 2) $$S=\frac{{\text{π}}^{2}k_{\text{B}}^{2}T}{3e}\frac{1}{R}\frac{dR}{dV_{\text{g}}}\frac{dV_{\text{g}}}{dE}\;{|}_{E=E_{\text{F}}}\;$$

where *T* is the sample temperature, *R* is the resistance, *E*_F_ is the Fermi energy and *k*_B_ is the Boltzmann constant. As the $\frac{dR}{dV_{\text{g}}}$ change signs as the gate voltage ($V_{\text{g}}$) across the CNP, *S* change signs every time when the voltages cross the yellow dashed lines in Figure 3A. This means when one side of the materials is kept close to CNP, the resistance *R* is dominated by the other side as the control gate is changing as shown in Figure 2B. Along the purple dashed line, although the Seebeck coefficient $S_{l}$ and $S_{\text{r}}$ is not switching sign, $S_{\text{l}}-S_{\text{r}}$ will switch sign. Therefore, the non-monotonic evolution of the photovoltage is proportional to $\left(S_{\text{l}}-S_{\text{r}}\right)$ and the 6-fold pattern in Figure 3A is direct evidence that the origin of the photovoltage is the photothermoelectric effect.

To clarify the different modes of the photodetector, we measured the spatial distribution of the photocurrent generated at the device. The photocurrent mapping process is described in STAR Methods and Figure S3B. As shown in Figures 3B and 3C, the photocurrent appears at the tunable p-n/n-p junction area indicated by the purple dashed lines. The maximum photocurrent value appears near the gap because the Seebeck coefficient contrast (*S*_l_ –*S*_r_) is maximized at the junction. The sign of photocurrent switches as the device changes from p-n to the n-p junction. If both the left gate and the right gate voltages are shifted to the charge neutrality point, we can also observe the photocurrent which comes from the Schottky junction between source/drain contacts (Cr/Au) and the tBLG. As shown in Figure 3D, the photocurrent at the gap area is minimized at the CNP and the photocurrent is observed near the Schottky junctions. So far, we have demonstrated a multimode photodetector enabled by changing the split-gate voltages.

**Figure 4:**
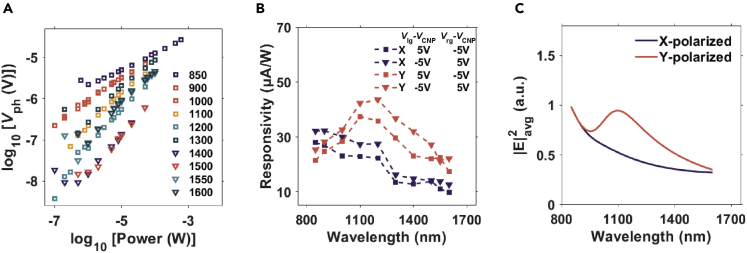
Linear dynamic range and the wavelength-dependent responsivity of the photodetector (A) Power-dependent photocurrent for different incident laser wavelengths ranging from 850 nm to 1,600 nm in the log-log scale. (B) Color-dependent responsivity for p-n/n-p junctions with incident polarization along the X and Y axis. (C) The averaged value of |E|^2^ at the tBLG interface within 1 μm across the split-gate gap for different polarizations.

To find the linear dynamic range, we measured the photovoltages for a few wavelengths ranging from 850 nm to 1,600 nm. The photovoltage changes linearly as a function of the incident laser power in the range from 0.1 μW to 100 μW. As shown in Figure 4A, we also noticed that the slopes of the power-dependent curves for different wavelengths are random possibly because the incident laser polarization was not the same for different wavelengths. To calibrate the wavelength-dependent responsivity, we fixed incident laser polarization along *X* or *Y* direction as defined in Figure 1B. We did the photocurrent mapping for p-n (*V*_lg_–*V*_cnp_ = *V*_cnp_ –*V*_rg_ = −5 V) or n-p (*V*_lg_–*V*_cnp_ = *V*_cnp_ –*V*_rg_ = 5 V) junctions for different wavelengths as shown in Figure S3. The laser powers are all kept at 100 mW. By averaging the photocurrent from 5 × 5 pixels near the same position, we plotted the photocurrent of the p-n and n-p junctions for both *X* and *Y* polarization as a function of incident laser wavelengths in Figure 4B. For both p-n and n-p junctions, the responsivity peak shows up at 1,150 nm if the laser polarization is along *Y* axis while the responsivity decreases as the laser wavelength changes from 850 nm to 1,600 nm if the polarization is along *X*-axis. To explain the observed polarization-dependent responsivity, we simulated electric field distribution using the finite-difference time-domain (FDTD) method. And the features of the responsivity are verified in Figure 4C. The responsivity (*R*) is proportional to the absorption (Abs∝s|E|^2^), which is the product of the conductivity σ and the square of electric field strength |E|^2^. As the conductivity of tBLG is almost flat except that the van Hove singularity may induce a small enhancement of absorption near 1,400 nm which is independent of polarization, the dominant photocurrent features here are mostly proportional to |E|^2^. The resonance of the electrical field at 1,150 nm for *Y*-polarized light is owing to a resonance mode formed in the multilayer structure as shown in Figure S4, which is very different from the electrical field distribution for *X*-polarized light. For our device geometry, the translational symmetry along the y axis is not perturbed and the tBLG is placed on top of the multilayer film structure (h-BN/air/SiO_2_/Si substrate) which forms a Fabry-Perot cavity for the *Y*-polarized light. But if the polarization is along the *X*-axis, the small gap will block most of the light leaking into the substrate and the electric field is concentrated mostly at the edges of gold films. Our observation and analysis explain the origin of the polarization-dependent responsivity for the near-infrared photodetector in the split-dual gate configuration.

In this study, we demonstrated a tBLG-based lateral p-n junction in the split-gate configuration which can perform as a multiple-mode near-IR photodetector controlled by electrostatic gate voltages. We confirmed that the photothermoelectric effect governs the photovoltage generation mechanisms. Previous studies have emphasized that adding a local heat absorber (Herring et al., 2014) as a substrate to increase the temperature gradient or using field enhancement structures (Xin et al., 2016) can increase the photocurrent generation, our results elucidate the origin of the polarization-dependent responsivity of the coplanar split-gate device and call attention to detailed structure designs to accurately control the polarization behavior. This study also provides an informative example for designing chip-integrated photodetectors in the future.

### Limitations of the study

The active layer of the photodetector was not fully encapsulated with h-BN because of a simpler fabrication procedure. However, in ambient condition, large electrostatic gate voltages can induce adsorption and even the electrochemical reaction at the surface. A better solution for a stable performance can be a fully encapsulated design.

## STAR★Methods

### Key resources table

**Table:** 

REAGENT or RESOURCE	SOURCE	IDENTIFIER
Other		

Graphenium Graphite	Manchester Nanomaterials	https://mos2crystals.com/product/graphenium-graphite/
h-BN	Manchester Nanomaterials	https://mos2crystals.com/product/hexagonal-boron-nitride-hbn/

Silicon Wafer (285 nm SiO_2_/Si)	LIJINGKEJI	https://item.taobao.com/item.htm?spm=2013.1.0.0.4942350fn2QF0M&id=5718821299&

### Resource availability

#### Lead contact

Further information and requests for resources and reagents should be directed to and will be fulfilled by the Lead Contact, Ya-Qing Bie (bieyq@mail.sysu.edu.cn) and Shaozhi Deng (stsdsz@mail.sysu.edu.cn).

#### Materials availability

All materials used and generated in this study will be made available on request from the lead contact with a completed Materials Transfer Agreement.

### Experimental model and subject details

#### Sample preparation

The fabrication of the device started by exfoliating the natural graphite crystal, h-BN from the *Manchester Nanomaterials* on the SiO_2_/Si chip. The van der Waals materials are stacked together using polycarbonate polymer under a microscope assisted by a homemade transfer setup. The layered materials were finally replaced on top of the prepatterned 2 nm/35 nm thick Ti/Au film split-gates. The split gates with an air gap of around 200 nm were prepared by E-beam lithography and thermal evaporation on the SiO_2_/Si substrate. The source-drain electrodes were overlayed via another E-beam lithography process afterward.

### Method details

#### Photovoltage measurement

The photovoltage and photocurrent measurements were taken by a homemade microscopic system equipped with a Janis ST500 cryostat. When the laser is focused on the junction surface, the photovoltage of the p-n junction is measured using a mechanic chopper at 387 Hz and the open-circuit voltages were collected using a lock-in amplifier SR830 as shown in Figure S3A.

#### Photocurrent measurement

As the laser incidents on the device surface, the photocurrent measurement at zero bias is collected with a current amplifier SR570 and the converted dc voltage is read by a National Instrument DAQ6218 as shown in Figure S3B. The photocurrent mapping is achieved by scanning a laser beam using a galvo mirror, then the photocurrent and the laser reflection signal (Gabor et al., 2011) are collected at the same time. Therefore, by comparing the two images, we can accurately determine from where the photocurrent comes. The incident laser source is the Fianium supercontinuum laser with a total output power of around 4 W. Each desired wavelength is filtered with high-quality bandpass filters from Thorlabs.

#### FDTD simulation

The FDTD method (FDTD solutions, Lumerical Inc) was employed to analyze the optical response of the device. The permittivity of gold was best fitted from the literature (Johnson and Christy, 1972) using the Drude model. The refractive index of Si was taken from experimental measurement (Vogt, 2015). h-BN was approximated as an anisotropic dielectric with refractive index (Pons-Valencia et al., 2019; Segura et al., 2018) *n*_xx_ = *n*_yy_ = 2.21 and *n*_zz_ = 1.72. The total-field scattered-field light source was incident from the air side. Perfectly matched layers were used to absorb the scattered radiation in all directions. In the simulation, a power monitor was used to record the electric field for integration. For the averaged electrical field in Figure 4C, since the translational symmetry is not broken along the y-axis, we only average the electrical field along the x-axis to mimic the light exciting area. The averaged width along the x-axis is 1 μm which is larger than the 200 nm wide gap. The averaged electrical field is described by the equation $|E_{avg}{|}^{2}=\frac{\int \left({\left|E_{x}\right|}^{2}+{\left|E_{y}\right|}^{2}\right)dx}{\int dx}$.

#### Estimation of the field-effect mobility

The field-effect mobility is estimated using the direct transconductance method (DTM) as discussed in literature (Zhong et al., 2015). The mobility can be described by the formula,

(Equation 3) $$\mu_{\text{DTM}}=g_{\text{m}}\frac{L}{WV_{\text{ds}}C_{\text{g}}}$$

where $g_{\text{m}}=\frac{\partial I_{\text{ds}}}{\partial V_{\text{g}}}$ is transconductance from the transfer curve shown in Figure 2D, the channel length *L* = 11.3 μm, the channel width *W* = 9.6 μm, the source-drain voltage *V*_ds_ is 1 mV, and the capacitance is calculated as $C_{\text{g}}=\epsilon \epsilon_{0}/t_{\text{BN}}$. The relative dielectric constant ε of h-BN is 3.76 as measured in the previous study (Laturia et al., 2018) and the thickness of the h-BN $t_{\text{hBN}}$ is 30 nm. The calculated maximum hole mobility and electron mobility is 3908 $\;{\text{cm}}^{2}/\text{ V}\cdot \text{s }$ and $2784\;{\text{cm}}^{2}/\text{ V}\cdot \text{s}$ using the DTM method.

## Data Availability

• Photocurrent data reported in this paper will be shared by the lead contact upon request. • This paper does not report original codes. • Any additional information required to reanalyze the data reported in this paper is available from the lead contact upon request.
