# Supplementary material for: A multimode photodetector with polarization-dependent near-infrared responsivity using the tunable split-dual gates control
Source: iScience. 2022 Sep 19;25(10):105164. doi: 10.1016/j.isci.2022.105164 (PMC9531171; doi:10.1016/j.isci.2022.105164)
Supplement: Document S1. Figures S1–S5 [file mmc1.pdf]

iScience, Volume *25*

Supplemental information

A multimode photodetector with polarization-dependent near-infrared responsivity using the tunable split-dual gates control

Zhou Zhang, Junxin Chen, Hao Jia, Jianfa Chen, Feng Li, Ximiao Wang, Shaojing Liu, Hai Ou, Song Liu, Huanjun Chen, Ya-Qing Bie, and Shaozhi Deng

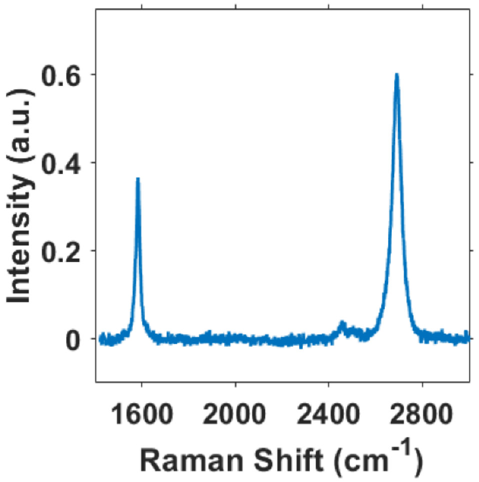

Figures S1.**The Raman spectrum of the tBLG sample with a twist angle near 4.5º**.Related to Figure 1.

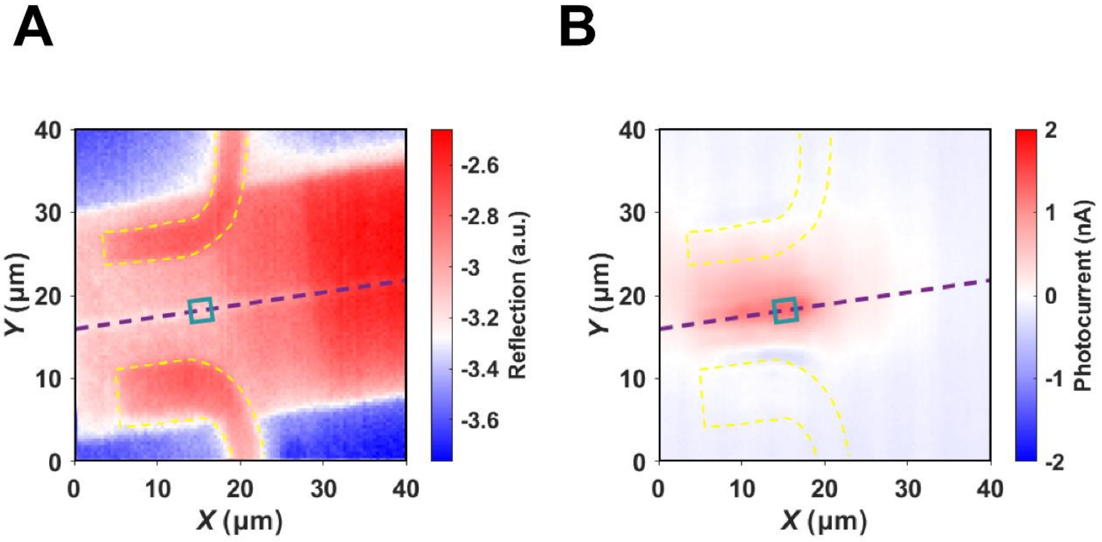

Figure S2.**The photocurrent mapping and the reflection mapping are measured simultaneously**. Related to STAR Methods.The incident laser wavelength is $\lambda = 900\ \mathrm{nm}$, and the optical power is 100 $\mu\mathrm{W}$. The gate voltages are set as $V_{lg} - V_{cnp} =$ 5 V; $V_{rg} - V_{cnp}$ = 5 V. The purple dashed lines indicate the location of the 200 nm gap. The cyan squares indicate the data position used to generate curves in **Figure 4B**. (**A**) the reflection mapping image (**B**) the photocurrent mapping image.

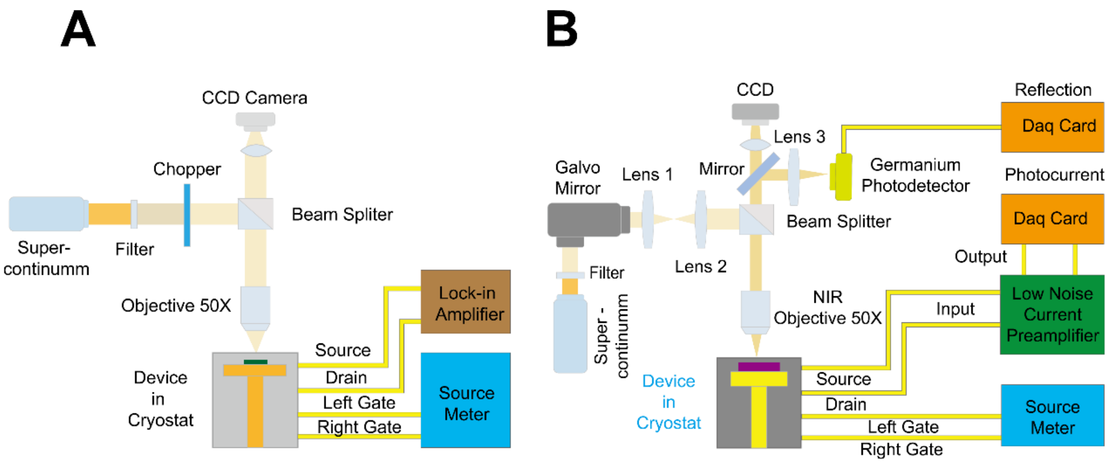

Figure S3.Schematics of the photovoltage and photocurrent measurement setups.Related to Figure 3. (**A**) photovoltage measurement (**B**) photocurrent mapping measurement.

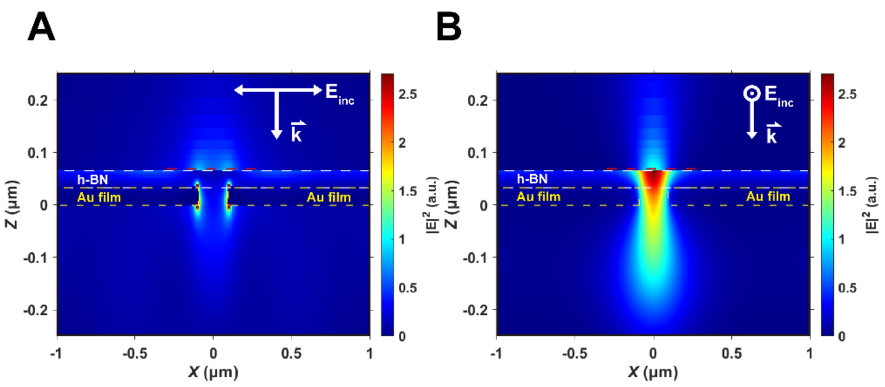

Figure S4.The distribution of the square of electric field strength ($|E|^2 = |E_x|^2 + |E_y|^2$) for different incident laser polarization in the X-Z plane.Related to Figure 4.(**A**) The incident laser polarization is along X-axis; the white long dashed lines show the 30 nm h-BN layer and the gold short dashed lines show the split gates made of gold; the red dashed line indicates the position of the tBLG layer. The electric field is enhanced near the gold film edge and two areas on the tBLG near the gold edges. (**B**) The incident laser polarization is along Y-axis and the electric field is mostly confined within the gap at the tBLG/h-BN interface.

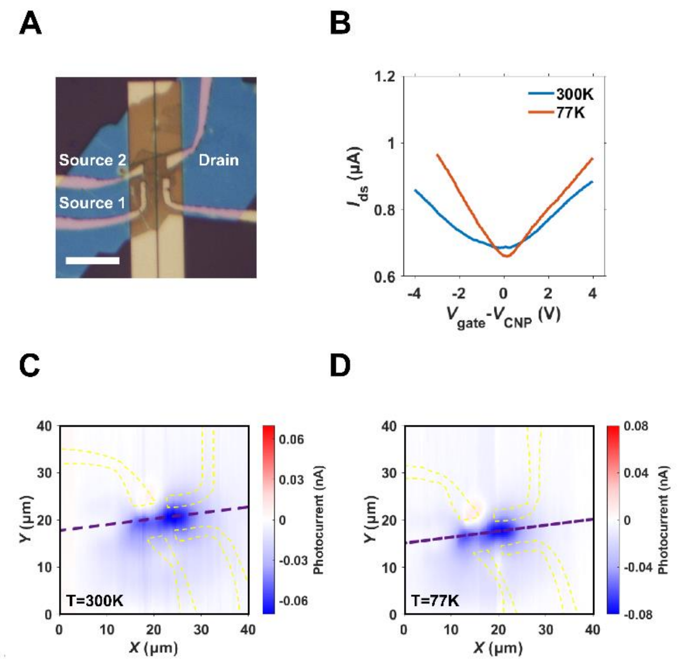

Figure S5.**Another sample was measured at both 77 K and 300 K**.Related to Figure 2.(A). The optical image of another split-dual gate sample. Here we fabricated four electrodes that contact with the tBLG. The scale bar is 10 μm; (B) The transfer curve of the transistor mode at 77 K and 300 K. The V_{ds} is set to 1 mV between the source 2 and drain electrodes. The mobility of the device increases as the temperature decreases to 77 K. (C) (D) The photocurrent mapping images at 300 K and 77 K in a vacuum. The wavelength of the incident laser is 850 nm and the power is 121 μW. The maximum photocurrent is -68 pA and -77 pA near the gap in (C) and (D), respectively. The left gate and right gate voltages are set as $V_{\mathrm{lg}} - V_{\mathrm{cnp}} = V_{\mathrm{cnp}} - V_{\mathrm{rg}} = 3.5\ \mathrm{V}$. The positive photocurrent near the drain electrode is due to the Schottky junction. The photovoltage will reach the maximum at 70 K according to the literature(Ma et al., 2014).
